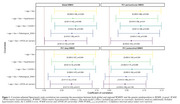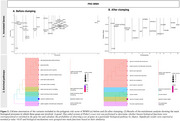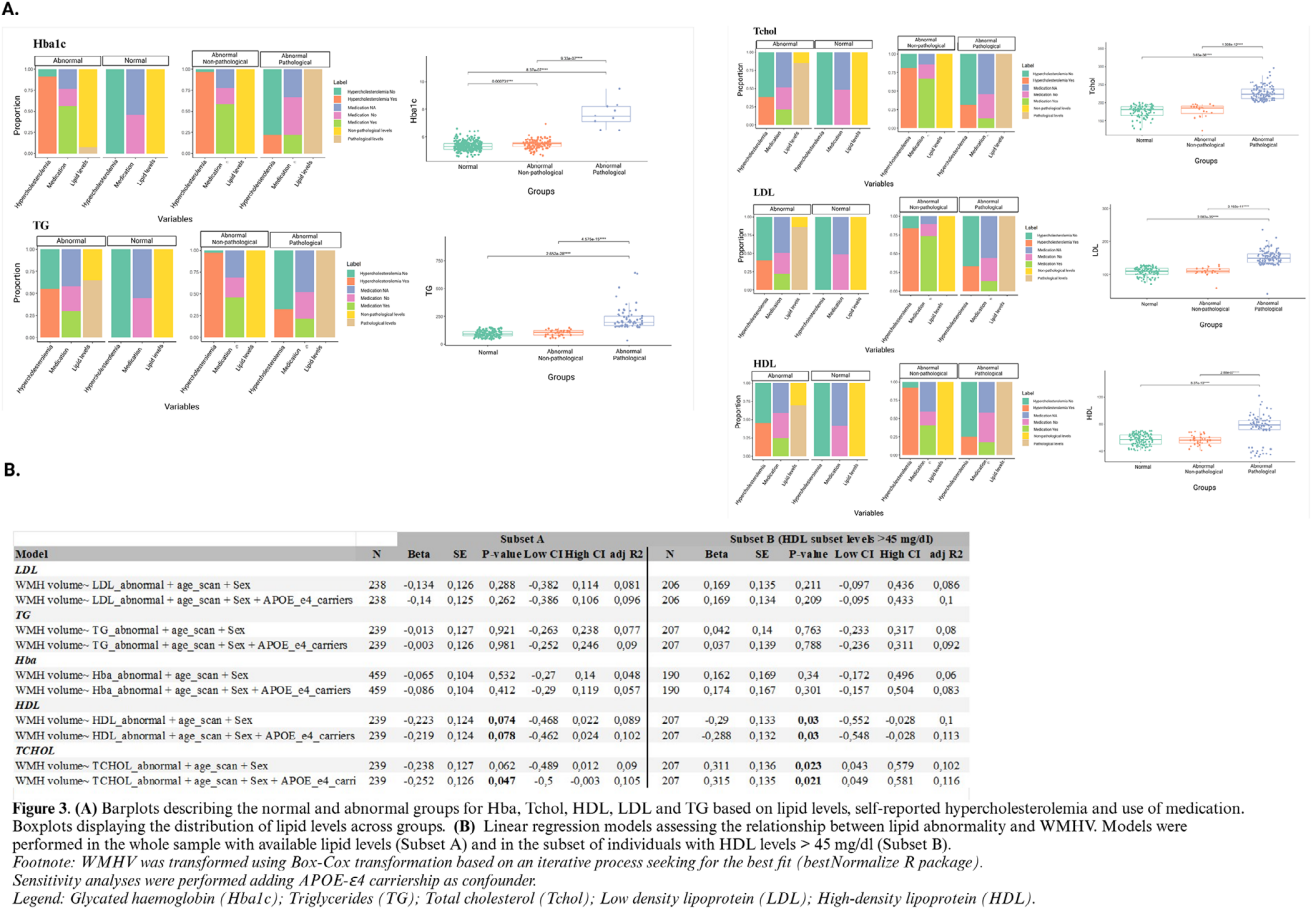# Polygenic risk of white matter hyperintensities and protective role of high‐density lipoprotein in cognitively unimpaired individuals at low risk for late life dementia

**DOI:** 10.1002/alz70856_107035

**Published:** 2026-01-08

**Authors:** Patricia Genius, Blanca Rodríguez‐Fernández, Carolina Minguillón, Anna Brugulat‐Serrat, Jordi Huguet, Manel Esteller, Carole H Sudre, Marta Cortes‐Canteli, Catarina Tristão‐Pereira, Ines Garcia‐Lunar, Arcadi Navarro, Juan Domingo Gispert, Natalia Vilor‐Tejedor

**Affiliations:** ^1^ Barcelonaβeta Brain Research Center (BBRC), Pasqual Maragall Foundation, Barcelona, Spain; ^2^ Centre for Genomic Regulation (CRG), Barcelona Institute of Science and Technology (BIST), Barcelona, Spain; ^3^ Hospital del Mar Research Institute, Barcelona, Spain; ^4^ Centro de Investigación Biomédica en Red de Fragilidad y Envejecimiento Saludable (CIBERFES), Madrid, Spain; ^5^ Global Brain Health Institute, San Francisco, CA, USA; ^6^ BarcelonaBeta Brain Research Center (BBRC), Barcelona, Spain; ^7^ Institució Catalana de Recerca i Estudis Avançats (ICREA), Barcelona, Spain; ^8^ Josep Carreras Leukaemia Research Institute (IJC), Badalona, Barcelona, Spain; ^9^ Physiological Sciences Department, School of Medicine and Health Sciences, University of Barcelona (UB), Barcelona, Catalonia, Spain; ^10^ Centro de Investigación Biomédica en Red Cancer (CIBERONC), Madrid, Spain; ^11^ Department of Neurodegenerative Disease, The Dementia Research Centre, UCL Queen Square Institute of Neurology, London, United Kingdom; ^12^ School of Biomedical Engineering and Imaging Sciences, King's College London, London, United Kingdom; ^13^ Hawkes Institute, University College London, London, United Kingdom; ^14^ Unit for Lifelong Health and Ageing, Department of Population Science and Experimental Medicine, University College London, London, United Kingdom; ^15^ Centro Nacional de Investigaciones Cardiovasculares (CNIC), Madrid, Spain; ^16^ Centro Internacional de Neurociencia Cajal (CINC), Consejo Superior de Investigaciones Científicas (CSIC), Madrid, Spain; ^17^ Cardiology Department, University Hospital La Moraleja, Madrid, Spain. CIBER de Enfermedades Cardiovasculares (CIBERCV), Madrid, Spain; ^18^ Institute of Evolutionary Biology (CSIC‐UPF) Universitat Pompeu Fabra, Barcelona, Spain; ^19^ AstraZeneca, Barcelona, Spain; ^20^ Department of Genetics, Radboud Medical University Center, Nijmegen, Netherlands

## Abstract

**Background:**

Cerebrovascular lesions, particularly white matter hyperintensities (WMH), are often found in middle‐aged individuals with a low cardiovascular risk profile. Understanding modifiable mechanisms leading to cerebrovascular disease is fundamental for implementing preventive strategies. This study aimed to elucidate the biological mechanisms underlying the presence of WMH in cognitively unimpaired (CU) middle‐aged individuals.

**Method:**

We included 1,072 CU participants from the ALFA study with a low cardiovascular risk profile for late‐life dementia based on the Cardiovascular Risk Factors, Aging, and Incidence of Dementia (CAIDE) I score. We assessed genetic predisposition to WMH using polygenic scoring (PRSWMH). Covariate‐adjusted Spearman's rank correlation tests evaluated the association between the PRSWMH and white matter hyperintensities volumes (WMHV). Partial correlations were adjusted for age and sex. Sensitivity analyses were performed adjusting the models for hypertension status, CAIDE score, WMH severity (Fazekas score 2), and *APOE*‐𝜀4 carriership. Next, enrichment analysis was conducted to identify the primary biological pathways (Gene Ontology; GO terms) enriched within the list of genes that confer higher risk of WMH (PRS‐annotated genes). Results lead to a final confirmatory analysis exploring the role of lipids as biomarkers of WMHV by examining the association of lipid abnormality (based on either self‐reported hypercholesterolemia, use of lipid‐modifying treatments, or pathological lipid levels) and WMHV in a nested cohort with available lipid measurements.

**Result:**

Genetic predisposition to WMH was associated with larger WMHV, even after controlling for confounders [Figure 1]. Lipid‐related biological processes were driving WMH genetic risk [Figure 2] and, remarkably, increased high‐density lipoprotein levels (HDL) acted as a protective factor against WMHV while higher total cholesterol (Tchol) appeared to be a risk factor [Figure 3].

**Conclusion:**

Lipid‐related mechanisms contribute to WMH in asymptomatic individuals at low risk for late‐life dementia based on their cardiovascular profile. In this population, high total cholesterol levels act as a risk factor for WMHV while high levels of HDL emerge as a protective factor. These individuals should be considered for lifestyle‐ and cholesterol‐lowering therapies to prevent dementia later in life.